# Medical and Physical Intelligence

**Published:** 1805-07-01

**Authors:** 


### MEDICAL AND PHYSICAL INTELLIGENCE.

[ FOREIGN AND DOMESTIC. ]

*The following Notice has been transmitted to* Dr. LETTSOM, *with a request from* Dr. STRUVE, *to circulate it as widely as possible.*

AUGURAPORY
Of the great and renowned old Magy,
ZOROASTER,
The " Clavis Artis,"
Which is contained in Thirty-nine Books, (which we have translated into German from the Arabian tongue, as faithfully as we were able) from the Year of the World 1996; rendered into German, Anno Christi 1236, by S. V. F. C. R. C.

*The following is an Extract from the Preface:*

" DURING my fifty years travels I never found this manuscript more than once in the Hebrew, Chaldæan, and Arabic, and then it was esteemed so great a treasure, that it could not have been purchased purchased for many thousand rixdollars. We were obliged to pay for such manuscripts, most of them being, like the old original, on copper-plates and dragon-skin, 50,000 rixdollars, or £. 8,350 sterling. And we never repented having purchased them so dearly, for it is most minutely specified and clearly demonstrated therein, both generally and particularly, how the most inveterate diseases are to be cured and subdued; we also found the universal key, as to what the Circulatum Majus and Minus is, of what it consists, how it is made and used, insomuch, that one has sufficient reason for concealing this as a secret Work, &c."

*Contents of Part the First:*

1 Book. Of the Maretz Aqua roris magalis and Aqua grandis.
2 —— Of the Patzlach or Alazagi.
3 —— Of the ♉. Abizar.
4 —— Of the Alacedach, Magnesia Stone, Algir or Pirtre Kies, and how the Philosopher's Stone is to be made thereof.
5 —— Of the Achium, which is found in the Euphrates and Nile and other rivers.
6 —— Of the Aladacha.
7 —— Of the Aajoc or Albativa, also denominated Pithon, the Mother of all Minerals.
8 —— Of the Aladron Microcosmi Adamici.
9 —— Of the Asophol Celuuilatel.
10 —— Of the Mineral Lachamai Asophol.
11 —— Of the Alneat and its secret workings.
12 —— Of the Hadit and Phosphoros and its secret Alazagi or Patzlach.
13 —— Of the Aladeipi and its secret workings.
14 —— Explains how the true *Hyelish* ♉ sapientiæ can be prepared from our *aboriginal* Chaos.
15 —— What is meant by the representation of the Triple-headed Dragon as the ♉, sulphur, and mercury from our old Albaon.
16 —— What is meant by the Red Lion clothed with the Sun.
17 —— How the Red Lion did eat and swallow our Pithon, and then united himself with him.
18 —— How the Phosphoros are to be metamorphosed into the pure Ayr by means of the Pithon, when it is made fixed and △, and is become white Tincture.
19 —— How our treble-headed Pithon is to be augmented and increased.
20 —— How the Algarisen prepared from the Alneat Hadit Phosphoros is to be united to the treble-headed Dragon.
21 —— Of the Albaon and its green and red Spirit; how this is to be united and made the greatest Tincture.
22 —— Of the green Dragon, and the manner of making a sweet °o therefrom.

23.

23 Book. Of the Algir Aladach, and the manner of preparing a Tincture on the Phosphoros in Ayr with it.

24 —— How a high Tincture is to be prepared by means of the Asophol Aceribe.

25 —— Of the true Circulatum Minus et Majus and its preparation.

26 —— Of Maretz; how Mars and Venus, or Man and Wife, as also the true ♉ naturæ is to be made from it, and how the real Universal can be produced by the union of these three.

27 —— Again treats of the Patzlach or secret Alazagi of Metals and Minerals

28 —— Of the true ♉ Abizar, and the manner of producing the △ of the Lord out of it.

29 —— Of the Lapis Algir or Sulphur Gravel; how the greatest Tincture is to be made by means thereof; and with it every disease to be cured, the Philosopher's Stone.

30 —— Of the Achium; how the greatest Tincture is to be made from it.

31 —— Of the Aladacha and its secret Alazagi.

32 —— Of Pithon, and the means of making out of it, in conjunction with Diana or Asophol and the ∴ Phosphoros, a Tincture.

33 —— Of Aladron, which comes from the microcosmick World, and the means of making with it, with the assistance of metals, an ∴ and Tincture.

34 —— Treats of Asophol Celuuilatel; how a true Alazagi can be made therewith, and with this Alazagi a ∴ and *Gluden*, out of which a powerful Tincture can be extracted.

35 —— In which the old Albaon is again mentioned, and the means of extracting from it first a red ∴ and then, with the assistance of the ☿ vit. a Tincture.

36 —— Of Alneat; how the Columba Dianæ and a sweet ♉ is to be made from it, and with both of these the greatest Tincture.

37 —— Of Hadit and Phosphoros, and therewith how to make a ∴ and a thick substantial Pithon, by means of which the greatest Tincture can be produced.

38 —— How an Aletur and an Arki are to be made from wine, as well as an Aladron, and how these three united, an Almes Aezi is to be extracted and prepared from metals, and therewith a powerful Tincture is to be made.

39 —— How to prepare by means of Asophol a blood-coloured ∴ for fermenting Tinctures with, and further how a ∴ can be prepared on Pithon by means of ☽ a.

*Contents of the Second Part:*

1 Book. Treats of the different means of preparing an Acharicho with the aid of Achium in the dry way.

2 —— How to prepare a Liq. out of the Aladron of a vine, in the former of which the Pirtre is discovered; furthermore, how other Pirtre can by a simple boiling be changed into Acaricho.

3.

3 Book How to ripen all unripe Achor, to prepare a Menstruum out of the Microcosmo and Vine, and, in short, how to extract all sorts of sulphura.

4 —— How to make a penetrating Tincture in the proper manner out of Albeta, which is artificial.

5 —— How to prepare an Acures out of Alneat, and with this Acures a red $\circ^{\circ}_{\circ}$ on Pithon

6 —— Directions for making an Aezi and Acaricho out of Alcubel with the Aladron of the Vine.

7 —— How a light Aezi can be made from the Asophol Aceribe with the aid of Abrasadum, and the greatest Tincture from a preparation of Asophol.

8 —— How an Albeta Adesia can be prepared from Schoschor, Pirtre, and Alneat, and an Aezi is to be extracted and made from this Albeta Adesia.

9 —— Teaches how to make a great Acaricho on Phosphoros in Ayr out of Acus Aschirz.

10 —— How man is to discover his Abimi Impressio Radischo, and to unite himself therewith, as also how he is to recognize his Abimi Fetagi Amaleach, and make use of it in a distinct manner.

*It is said in the Preface to Part the Second;*

" We were obliged to spare no trouble or expence in procuring the Figures in the Plates to the last 10 Books, because great Mysteries, whereof not a syllable is mentioned in the other Books, are concealed in them, such as the accurate description of the dry Way, the means of preparing in a short time a Tincture with the assistance of *flint*, by melting, which few Authors take any notice of: For Kiesel *(flint)* is a hidden ⊖ alcali, or properly speaking, metakorum, which contains all sulphura within itself, and therewith becomes a Tincture." The original M. S. contains 384 Folio Pages and about 60 painted Drawings of Figures, Characters, &c.; the Text is legible, and the whole Work free from blemish. This M. S. certainly an unique of its kind, is marked No. 1. to shew that it occupied the first place in the *hermetic* Library, whence it came. It is evident from some Remarks in it, that its doctrines have been proceeded upon with success; among others, is this: " It is true, I did not obtain more than 4 oz. from a cwt. but it was excellent, and do not all Kiesel give as much ?" The key is added in the margin throughout the Work. This Manuscript, which is to be sold to the highest Bidder, can be valued, without exaggeration, at 80m. rixdollars, or from 13 to 14,000*l.* sterling. The Term of bidding shall last half a year, and all propositions are to be addressed to me as under, free of postage. After receipt of payment, the MS. is to be forwarded to the Buyer in whatever manner he may think fit. Goerlitz, in Upper Lusatia, November, 1804.

I. G. BRAUN, Deacon of St. Peter's.

To

To Dr. Lettsom.

Dr. Struve requests, honoured Sir, that you will make this Work public through the medium of the English Journals. Perhaps, Sir, you may have an opportunity of fixing the attention of some lover of this science on this hermet MS. and therefore permit me to entreat your kind co-operation. I could wish any persons, desirous of treating, might, in the first place, communicate their Propositions to you, and that you would afterwards be so kind as to advise Dr. Struve of the Communication.

I. G. Braun.

---

A Correspondent, to whom the Editors are indebted for several valuable papers, has requested the insertion of the following

QUERIES.

1. Are not the phenomena which result from the effects of simple vaccination confined to the cuticle and rete mucosum?

2. Does not this view of vaccination, possibly, account for the absence of fever and eruption?

3. When constitutional affection does occur, is not this sympathetic?

4. Is not the sequela after vaccination ill described by the term cicatrix?

5. Does not that which we have been accustomed to call cicatrix denote previous suppuration, and loss of the cutis vera at least?

6. Is not the only loss or sloughing in the process of vaccination, that of the rete mucosum?

7. Do not the rete mucosum and cutis vera both generally slough in the small-pox?

8. When the vaccine principle reaches accidentally the blood vessels, is not this an error loci, a cause of constitutional affection, and source of irregularities and anomalies?

9. Cannot the areola be explained consistently with this view of the cow-pock?

N. B. These Queries are very respectfully submitted to Mr. Ring, Dr. Walker, and your other Correspondents.

---

Dr. Trotter, of Newcastle, is preparing for the Press, An Enquiry into the increasing Prevalence, Prevention and Treatment of those Diseases, commonly called, Nervous, Bilious, Indigestion, &c. This Work, which has engaged his attention for a number of years, is purely practical, though he attempts a General Doctrine on the subject. Certain it is, in the present day, complaints of this class form by far the greatest proportion of all the diseases which come under the knowledge of the physician. It must therefore be not only curious but useful, to contrast the health of the savage state, and the days of chivalry, with the modern condition of commercial society. The influence of these diseases is to be observed in the characters of nations nations as well as families, and claim alike the attention of the patriot and parent. The effects which mercury, opium, tea, &c. have in their production, are duly considered; and what relates to the cure, is founded on the experience of some thousands of cases, diversified by character, sex, age, rank, employment, climate, &c. and on more extensive intercourse with the different conditions of mankind, than has usually fallen to the lot of one physician.

Non his juventus, orta parentibus

Infecit aquor sanguine punico.

---

Dr. ADAMS, the new Physician to the Small-Pox Hospital, whilst preparing his second edition of "Morbid Poisons," has found time to put together a small Tract, which he calls, "Answers to all the Objections hitherto made against Cow-pox." It is written in a popular style, printed on a cheap paper, and sold for the Benefit of the Hospital.

A Treatise will appear in a few days on the Process employed by Nature in suppressing the Hæmorrhage from divided and punctured Arteries, and on the Rise of the Ligature, concluding with Observations on Secondary Hæmorrhage; the whole deduced from an extensive series of experiments, and illustrated with fifteen plates, by Dr. Jones, of Barbadoes.

A Treatise on the Anatomy of the Human Ear, by Mr. J. C. SAUNDERS, demonstrator of practical anatomy at St. Thomas's Hospital, and surgeon of the London Dispensary for Diseases of the Eye and the Ear, will shortly make its appearance. It will be illustrated by a series of engravings, which represent the organ precisely of its natural size. The engravings will be executed in the best style, and will be made from drawings taken by an excellent artist from dissections of the recent ear. The engravings will shew, in succession, the different parts exactly in their natural position, and, taken together, will form a complete demonstration of this complicated organ.

Dr. S. H. JACKSON has in the press, an Inquiry into the Nature of the Disease which so lately prevailed at Gibraltar, with Remarks on Epidemic Fevers in general.

Dr. HARTY will publish in the course of the present month, a work entitled, Observations on the simple Dysentery and its combinations, containing a review of the most celebrated authors who have written on that subject, and likewise an investigation into the Source of Contagion in that and some other Diseases.

The late Dr. GARNETT's Zoonomia, or popular Lectures on the Laws of Organic Life in Health and Diseases, is in the course of delivery to the subscribers.

Dr. DENNISON and Dr. SQUIRE will on Tuesday, the 9th of July, begin a Course of Lectures on the Principles and Practice of Midwifery, and the Diseases of Women and Children.

